# Cellular Models of Aggregation-dependent Template-directed Proteolysis to Characterize Tau Aggregation Inhibitors for Treatment of Alzheimer Disease*[Fn FN2]

**DOI:** 10.1074/jbc.M114.616029

**Published:** 2015-03-10

**Authors:** Charles R. Harrington, John M. D. Storey, Scott Clunas, Kathleen A. Harrington, David Horsley, Ahtsham Ishaq, Steven J. Kemp, Christopher P. Larch, Colin Marshall, Sarah L. Nicoll, Janet E. Rickard, Michael Simpson, James P. Sinclair, Lynda J. Storey, Claude M. Wischik

**Affiliations:** From the ‡ School of Medicine and Dentistry, University of Aberdeen, Aberdeen AB25 2ZP, United Kingdom,; § TauRx Therapeutics Ltd., Singapore 068805, and; the ¶ Department of Chemistry, University of Aberdeen, Aberdeen AB24 3UE, United Kingdom

**Keywords:** Alzheimer Disease, Cell Biology, Protein Aggregation, Tau Protein (Tau), Tauopathy, LMTX, Prion-like Processing, Methylthioninium

## Abstract

Alzheimer disease (AD) is a degenerative tauopathy characterized by aggregation of Tau protein through the repeat domain to form intraneuronal paired helical filaments (PHFs). We report two cell models in which we control the inherent toxicity of the core Tau fragment. These models demonstrate the properties of prion-like recruitment of full-length Tau into an aggregation pathway in which template-directed, endogenous truncation propagates aggregation through the core Tau binding domain. We use these in combination with dissolution of native PHFs to quantify the activity of Tau aggregation inhibitors (TAIs). We report the synthesis of novel stable crystalline leucomethylthioninium salts (LMTX®), which overcome the pharmacokinetic limitations of methylthioninium chloride. LMTX®, as either a dihydromesylate or a dihydrobromide salt, retains TAI activity *in vitro* and disrupts PHFs isolated from AD brain tissues at 0.16 μm. The *K_i_* value for intracellular TAI activity, which we have been able to determine for the first time, is 0.12 μm. These values are close to the steady state trough brain concentration of methylthioninium ion (0.18 μm) that is required to arrest progression of AD on clinical and imaging end points and the minimum brain concentration (0.13 μm) required to reverse behavioral deficits and pathology in Tau transgenic mice.

## Teaser

**Background:** Tau aggregation inhibitors could treat Alzheimer disease.

**Results:** Stable reduced forms of leucomethylthioninium (LMTX®) are active in blocking prion-like Tau aggregation in novel cellular models.

**Conclusion:** The intracellular *K_i_* (0.12 μm) is comparable with brain levels required for clinical benefit.

**Significance:** LMTX® could treat Alzheimer disease.

## Introduction

Alzheimer disease (AD)^3^ is an irreversible, neurodegenerative disorder characterized by the formation of neurofibrillary tangles. These tangles were discovered by Alzheimer (1) and are made up of pathological paired helical filaments (PHFs) composed predominantly of a truncated 100-amino acid fragment of the microtubule-associated protein Tau (2). Numerous studies have confirmed correlations between the quantity of aggregated Tau, spread of neurofibrillary tangle pathology, extent of clinical dementia, and functional molecular imaging deficits (3–8). There is increasing interest in the possibility of developing a Tau-based approach to treatment of AD.

We previously reported an assay for Tau-Tau binding through the repeat domain in which we demonstrated that the repeat domain has the ability to define a template-directed truncation of full-length Tau to reproduce a proteolytically stable species characteristic of the PHF core in AD and that the process could be propagated through stepwise binding and digestion cycles (9). We now report the development of two cell-based assays that permit demonstration of prion-like processing of Tau in the more physiological context of the cell. Compounds which have Tau aggregation inhibitor (TAI) activity in cell-free assays block template-directed truncation within cells with approximately the same rank order, showing that the process is aggregation-dependent. These models confirm that Tau protein, in the absence of any post-translational modification or covalent cross-linking, is inherently able to form proteolytically resistant aggregates through the repeat domain in a physiological environment and that this process recruits normal Tau to undergo template-directed proteolytic truncation to form characteristic neofragments, including the core Tau unit of the PHF.

**FIGURE 1.**
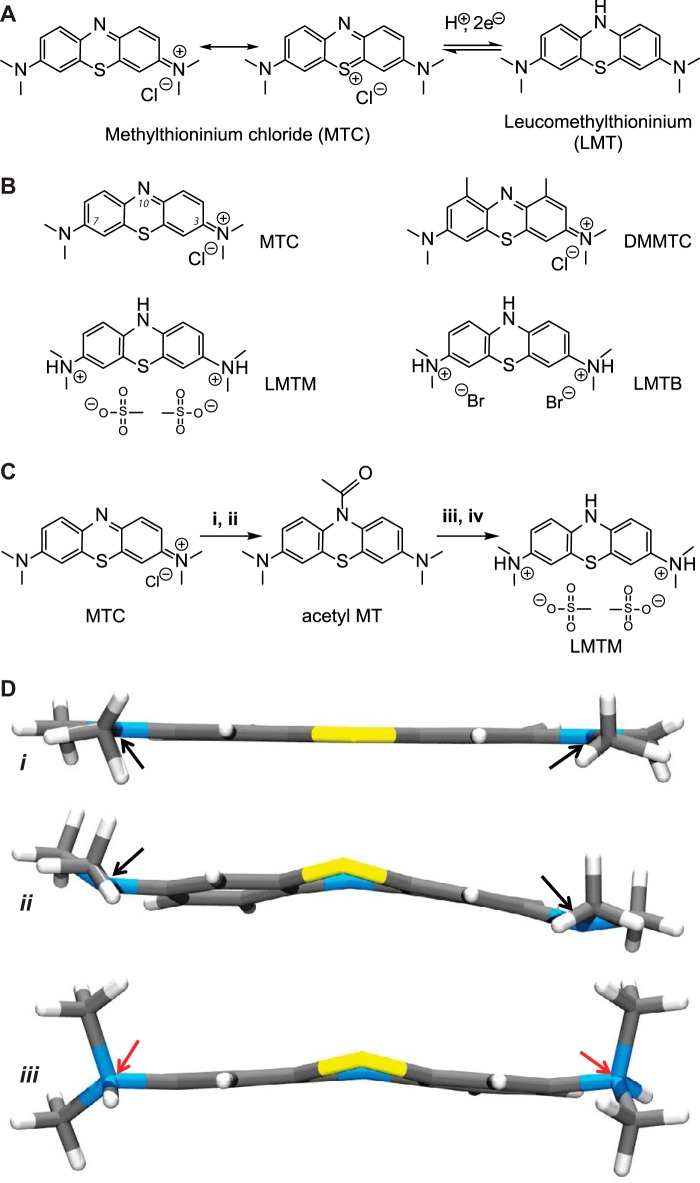
**Characterization of compounds.** *A*, redox conversion of MTC to leucomethylthioninium. *B*, structures of compounds tested in assays: MTC, DMMTC, LMTM, and LMTB. *C*, scheme for synthesis of LMTM from MTC. *i*, N_2_H_4_·H_2_O, Et_3_N, CH_3_CN, N_2_, 70 °C, 1 h; *ii*, Ac_2_O, N_2_, 95 °C, 2 h; *iii*, methanesulfonic acid, H_2_O, 78 °C, 2.5 h; *iv*, EtOH. Details are provided under “Experimental Procedures.” *D*, x-ray structures of MTC (22) (*i*), MW (*ii*), and LMTM (*iii*). The nitrogen atoms at positions 3 and 7 are planar in the case of MTC or pyramidal in the case of MW (*black arrows*) and tetrahedral for LMTM (*red arrows*).

The primary motivation in developing the cell assays that we report has been to aid the optimization of TAIs, as an intermediate between primary screening and testing in transgenic animal models, prior to clinical testing. Methylthioninium chloride (MTC; commonly known as “methylene blue”), the first reported TAI, reverses the proteolytic stability of PHFs isolated from AD brain tissues *in vitro* without disrupting normal Tau-tubulin interactions (9). MTC is a stable heterocyclic ionic molecule, which may exist in equilibrium, depending on environmental conditions (*e.g.* pH, level of oxygenation, and the presence of oxidizing or reducing agents), with its oxygen-sensitive redox couple, leucomethylthionium (LMT; also known as “methylene white” (MW)) (Fig. 1*A*). A phase 2 clinical trial in mild/moderate AD identified a dose of 138 mg of MT/day, administered as MTC, as the minimum effective dose on clinical and imaging outcomes at 24 weeks (10). However, a dose of 228 mg of MT/day had either no efficacy or limited efficacy on the same end points, due to limitations in the ability to absorb the MT in its cationic form (11). This is because MTC behaves effectively as a pro-drug that requires active reduction to the uncharged LMT form in the gut to permit absorption by passive diffusion and subsequent distribution to the brain (11). As a result of dose-dependent limitations in this process, the 138 mg of MT/day dose was simply the highest available dose, and testing for efficacy and safety of higher doses of MT is not feasible clinically using MTC (10).

In order to overcome this limitation, we report the synthesis of novel chemical entities (which we denote LMTX®) in which the reduced LMT form can be provided directly as a stable anhydrous crystalline salt suitable for pharmaceutical development. We use the new cell assays to analyze different diprotic salt forms of LMT and show that they retain TAI activity within the physiological milieu of the cell. We use the assays to determine that the intracellular *K_i_* for activity of MT at the Tau aggregation site is 0.12 μm. This value is close to the P_50_ value for disaggregation of PHFs isolated from AD brain (0.16 μm), the minimum brain concentration (0.13 μm) required to reverse behavioral deficits and reduce Tau pathology in Tau transgenic mice (12), and the estimated steady state trough brain concentration of MT and its pharmacologically active demethyl derivatives (0.18 μm) at the minimum effective dose found to be required for arresting the progression of AD (11).

## EXPERIMENTAL PROCEDURES

### 

#### 

##### Compounds

10-Acetyl-*N*,*N*,*N*′,*N*′-tetramethyl-10*H*-phenothiazine-3,7-diamine was synthesized and used as the starting material for the synthesis of *N*,*N*,*N*′,*N*′-tetramethyl-10*H*-phenothiazine-3,7-diaminium dibromide (which we denote leucomethylthioninium dihydrobromide for convenience; LMTB) and *N*,*N*,*N*′,*N*′-tetramethyl-10*H*-phenothiazine-3,7-diaminium bismethanesulfonate (which we denote leucomethylthioninium dihydromesylate; LMTM). Detailed syntheses can be found in the supplemental material. MTC was purchased from Simpsons (Gwent, UK). 1,9-Dimethyl methylthioninium chloride (listed as “1,9-dimethylmethylene blue” and which we refer to as DMMTC) was purchased from Serva. The structures of these compounds are shown in Fig. 1*B.*

##### Polymorphism Analysis of LMTM

A polymorphism analysis was conducted by Solvias AG (Kaiseraugst, Switzerland) to investigate the predisposition of LMTM to show polymorphism or hydrate formation. Two types of crystallization experiment were conducted: suspension equilibrations, generally considered the most reliable way to discover stable polymorphic forms, and cooling crystallizations, which increase the probability of identifying kinetically preferred but thermodynamically metastable polymorphic forms from highly saturated solutions. Both FT-Raman spectroscopy and powder x-ray diffraction were used to analyze samples (13).

##### PHFs and Tau Proteins

PHFs were purified from AD brain as described previously (2). Fragmented PHFs, termed ABCsup (14), were used for the primulin-binding P_50_ assay. Recombinant Tau fragments dGA and dGAE, corresponding to amino acids 297–390 and 297–391, respectively, of the largest human Tau isoform in the central nervous system (15), were expressed in bacteria. These proteins were purified from bacterial cell lysates using P-11 phosphocellulose ion exchange chromatography (16).

##### Primulin-binding Assay (P_50_)

The disruption of PHFs was monitored by the effect of compounds on enhanced PHF-dependent fluorescence measured using primulin binding to PHFs. Samples were assayed in 96-well white plates (100-μl volumes) in the presence of primulin (1 μm) and test compounds at appropriate concentrations. The fluorescence was measured in a Varian Carey Eclipse fluorescence spectrometer, and the excitation spectrum was measured with the emission wavelength at 480 nm. Peak fluorescence at an excitation wavelength of 420 nm was measured from spectra after correction for the signal measured in the absence of PHFs. The P_50_ was calculated as the concentration of compound at which the fluorescence is reduced to 50% of the value in the absence of compound.

##### Tau-Tau Binding Assay (B_50_)

Compounds were tested for inhibition of Tau-Tau binding using an ELISA-based assay (9). The Tau fragment dGA, at 1 μm in 50 mm carbonate buffer (pH 9.6), was adsorbed to the surface of 96-well poly(vinyl chloride) plates. After blocking with 2% dried milk powder in phosphate-buffered saline (PBS), dGAE (0.15 μm in 25 mm K-PIPES, 50 mm NaCl, 0.05% Tween® 20, 1% fish skin gelatin, pH 6.8) was added with test compounds. All incubations were at 37 °C for 60 min. Bound antibody was detected as described previously (9). The B_50_ value was calculated as the concentration of inhibitor at which specific binding was reduced to 50% of the value in the absence of inhibitor.

##### Inducible Tau(1–441) (hTau40) Vector

A cell line expressing full-length human Tau was constructed using the LacSwitch® (Stratagene) system in which Tau is expressed under control of the *lac* repressor protein. 3T6 cells (ECACC number 86120801 mouse Swiss albino embryo fibroblasts) were transfected, by electroporation, with the p3′SS plasmid, which encodes the *lac* repressor protein, and colonies were selected for hygromycin resistance (3T6H cells). A NotI cloning site was introduced into hTau40 cDNA to allow cloning of Tau into the pOPRSVICAT vector by PCR-based mutagenesis using the following primers: forward primer, 5′-gtcgactctagaggcggccgcatggctgagccccggcaggag-3′; reverse primer, 5′–actcttaagggtcgcggccgctcacaacaaaccctgcttggccag-3′. The insert was ligated into the vector, and the correct orientation of the inserts was confirmed by restriction digest mapping.

The pOPRSVT40 plasmid was transfected by electroporation into 3T6H cells, and clones were selected for resistance to G418. The expression of full-length hTau40 was induced using isopropyl β-d-1-thiogalactopyranoside (IPTG).

##### Expression of hTau40 with SSTau Constructs

The expression system used for production of membrane-associated Tau fragments is based on the observation that rabbit globin mRNA is translated on membrane-bound ribosomes if the signal sequence (SS) of rat albumin is inserted at the 5′ end of the cDNA (17). The vector described previously for expression of globin was modified to incorporate fragments of Tau coding sequence downstream of the signal sequence while at the same time maintaining the globin 3′-untranslated region.

Three vectors expressing truncated forms of Tau protein (residues 296–390, 190–441, and 190–390) and referred to as SSTau(296–390), SSTau(190–441), and SSTau(190–390), respectively, were constructed. PCR-based mutagenesis on pcKSSGG (17) was used to introduce an AgeI site at the globin start codon to make pcKSSGGAgeI (forward primer, 5′-gccttttcaccggtgcatctgtcca-3′; reverse primer, 5′-tggacagatgcaccggtgaaaaggc-3′). For *pcKSSTau(190–441)*, PCR-based mutagenesis on hTau40 introduced an AgeI site at nucleotide 1128 of hTau40 (forward primer, 5′-tctggtgaaccggtaaaatcagggg-3′; reverse primer, 5′-cccctgattttaccggttcaccaga-3′). The fragment was cut with EcoRI beyond the Tau stop codon, blunt-ended, cut with AgeI, ligated into pcKSSGGAgeI, cut with BamHI, blunt-ended, and then cut with AgeI to make pcKSSTau(190–441). The ligation process results in Lys-190 becoming the start of the Tau sequence. For *pcKSSTau(190–390)*, PCR-based mutagenesis on pcKSSTau(190–441) introduced a BamHI site and a stop codon at nucleotide 1740 (which terminates the Tau sequence at Ala-390) (forward primer, 5′-cggggcgtatccgtacaagtcg-3′; reverse primer, 5′-cgacttgtacggatcctacgccccg-3′). The fragment was cut with AgeI and BamHI and ligated into pcKSSGGAgeI at the same sites to make pcKSSTau(190–390). For *pcKSSTau(296–390)*, PCR-based mutagenesis on pcKSSTau(190–390) introduced an AgeI site at nucleotide 1450 of pcKSSTau(190–390) (forward primer, 5′-ggctcaccggttaatatcaaac-3′; reverse primer, 5′-gtttgatattaaccggtgagcc-3′). The fragment was cut with AgeI and BamHI and ligated into pcKSSGGAgeI at the same sites to make pcKSSTau(296–390). These constructs were created using the expression vector pcDNA3.1, which carries the G418 resistance marker. All were subcloned into the pcDNA3.1/Zeo vector (Invitrogen, Leek, Netherlands), which contains the Zeocin resistance marker, for transfection into the inducible hTau40 cell line.

##### Expression of hTau40 with Constitutive Tau(295–391)

For construction of the plasmid pZeo(295–391), designed to express protein corresponding to a truncated fragment of Tau (residues 295–391), EcoRI and BamHI cloning sites were introduced by PCR amplification of hTau40 cDNA (forward primer, 5′-cggaattccaccatggataatatcaaacacgtcccg-3′; reverse primer, 5′-cgcgggatcctcactccgccccgtggtctgtcttggc-3′). The constructs were subcloned into pcDNA3.1/Zeo(−) for transfection into the hTau40 cell line.

##### Cell Culture

Cells were grown in Dulbecco's modified Eagle's medium (with Glutamax I, pyruvate, 4.5 g/liter glucose; Life Technologies), supplemented with 10% fetal calf serum (Helena BioSciences), 50 units/ml penicillin, 50 μg/ml streptomycin, and further antibiotic as appropriate for the selection and maintenance of the relevant plasmids. Hygromycin (200 μg/ml) and G418 (500 μg/ml) were included for both selection and maintenance of the hTau40 cell line, and 400 or 200 μg/ml Zeocin was used for selection or maintenance, respectively, of SSTau(190–441), SSTau(190–390), SSTau(296–390), and pZeo(295–391). Cells were grown at 37 °C, in a humidified atmosphere containing 5% CO_2_.

##### Cell-based Tau Aggregation Assay (EC_50_)

**FIGURE 2.**
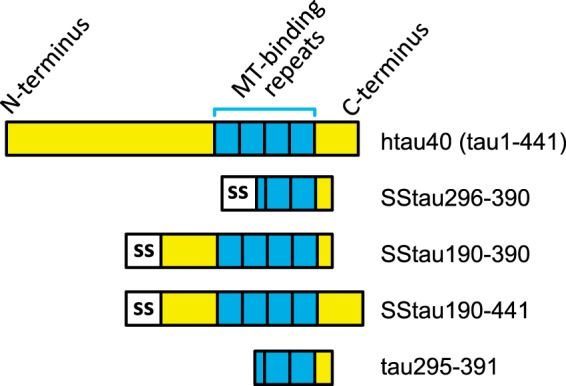
**Tau constructs used in cell models.** Full-length Tau (hTau40; amino acid residues 1–441) was used in an inducible vector system described under “Experimental Procedures.” The hTau40 cell line was then transfected with truncated Tau fragments that are expressed constitutively to generate two different models: either with one of a series of SSTau constructs of various lengths or with truncated Tau(295–391) without the SS.

The cell-based Tau aggregation assays used the cell lines described above, which express inducible full-length hTau40 in combination with constitutive expression of one of the Tau fragments selected from SSTau(190–441), SSTau(190–390), SSTau(296–390), and Tau(295–391) (see Fig. 2). Expression of hTau40 was induced by the addition of IPTG (10–50 μm); in the case of cells expressing the SSTau fragments, their expression was increased by the addition of sodium butyrate (5 mm), a nonspecific enhancer of protein expression (18, 19).

For testing of compounds, cells were incubated with compounds in 24-well plates, and after 24 h, IPTG was added to the medium. After overnight incubation, the medium was removed, and cells were washed with PBS and then taken up with Laemmli buffer. Samples were separated by SDS-PAGE, transferred to PVDF membrane, and reacted with mAb 7/51 antibody (14, 20). Bound Tau antibody was detected using enhanced chemiluminescence. Compounds were typically tested at four concentrations, each in triplicate, with all samples being separated on a single gel. To control for any variability in expression of hTau40, we use the ratio of the intensities of the truncated 12 kDa to full-length hTau40 protein bands. The values, normalized to those obtained for control samples in which there had been no test item, were plotted against compound concentration. The EC_50_ value was determined as the concentration at which the ratio was 50% of the value obtained in the absence of compound.

##### Tau Antibodies

The following Tau antibodies were used: mAbs 7/51 (epitope in microtubule-binding repeat domain (14, 20)); 27/499 (N-terminal epitope in residues 14–26 (9)); 27/342 (epitope in residues 208–251 (9)); and T46 (C-terminal epitope in residues 395–432; Invitrogen).

##### Cellular Toxicity

Toxicity was determined using the Cytotox 96-well kit (Promega), according to the manufacturer's instructions. The LD_50_ was determined as the concentration in the assay at which the absorbance at 490 nm was decreased by 50%.

##### Immunofluorescence

3T6 cells expressing Tau were grown on glass coverslips for microscopy. Cells were fixed either in 0.3% glutaraldehyde containing 0.1% Triton® X-100 or in paraformaldehyde for 20 min followed by extraction with 0.2% Triton® X-100. In some cases, cells were extracted with saponin (0.5% in 80 mm K-PIPES, 1 mm MgCl_2_, 1 mm EGTA, 30% glycerol, pH 6.8) for 2 min and then fixed in 0.3% glutaraldehyde in the same buffer for 10 min. Saponin (0.5%) was included in subsequent antibody incubations and washes. Saponin is a relatively mild detergent that leaves internal membranes intact, whereas Triton® X-100 will expose the lumenal surface of internal organelles. Cells were then labeled by incubation for 1 h at room temperature with mAb 7/51 (hybridoma supernatant fluid diluted 1:10 in PBS containing 0.05% Tween® 20). Antibody was detected using goat anti-mouse FITC conjugate. The rat mAb YL1/2 was used for visualization of microtubules (21). Cells were examined by fluorescence microscopy.

## RESULTS

### 

#### 

##### Synthesis and Manufacture of LMT as Pure Stable Crystalline Salts

We report the development of an industrially scalable synthesis of LMT salts as novel chemical entities (LMTX®) using crude MTC as the starting material (organic purity less than 97% and metal impurity in excess of 20 μg/g). The synthetic scheme for the dihydromesylate salt, as an example, is shown in Fig. 1*C*, whereas details of the synthesis are provided in the supplemental material. When scaled up, the final crystalline product was isolated in 84% yield on a 90-kg industrial synthesis with a 99.7% organic purity and heavy metal content within the permitted European Pharmacopoeia limits specified for MTC (see supplemental Table S1).

The single crystal, x-ray structural determinations of MTC (22); the oxygen-sensitive, uncharged LMT (MW) (stored under argon); and the oxygen-stable, protonated LMTM are compared in Fig. 1*D*. The core ring system of MTC is planar, as expected for a completely delocalized system; in contrast, MW and LMTM have non-planar central heterocyclic rings, consistent with the reduced form. The x-ray crystallography for LMTM confirmed that the nitrogen atoms at positions 3 and 7 had tetrahedral geometry and hence were protonated. Protonation of the nitrogen atoms contributes to both the geometry of LMTM and its stability in an oxygen atmosphere and differentiates it from MW, which has trigonal pyramidal nitrogen geometries. These structures distinguish the protonated LMTM from MW and confirm that LMTM is a distinct chemical entity.

Of the LMTX® salt forms investigated (dihydrobromide and dihydromesylate), LMTM exhibited optimal characteristics in terms of crystal properties, purity (99.7%), stability to oxidation, and process scalability. LMTM is substantially more water-soluble than MTC (1,700 and 18 mg/ml, respectively) and exists as a single polymorph (see Raman spectrum and x-ray diffraction pattern of the LMTM polymorph in supplemental Figs. S1 and S2). The LMTM polymorph does not change on storage at 30 °C and 65% humidity over 24 months (see supplemental Table S2).

##### Dissolution of PHFs Isolated from AD Brain Tissues

**FIGURE 3.**
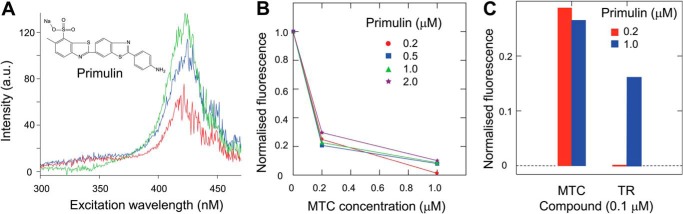
**Disruption of PHFs by MT.** *A*, PHF-dependent fluorescence excitation spectra (emission wavelength, 480 nm) measured with primulin and increasing concentrations of PHFs (2, 5, and 10 μl of PHFs, indicated by *red*, *blue*, and *green profiles*, respectively). The spectra shown have been corrected for signal measured in the absence of PHFs. *B*, PHF-dependent fluorescence at increasing concentrations of MTC in the presence of primulin at 0.2–2 μm, as indicated, with 10 μl of PHFs. The fluorescence signal measured at 420-nm excitation and 480-nm emission was corrected by subtraction of the signal measured in the absence of PHFs and normalized to the signal measured in the absence of MTC. There was no difference in MTC inhibition of fluorescence over the range of primulin concentrations tested. *C*, thiazine red (*TR*) at 0.1 μm binds to PHFs as a ligand and inhibits fluorescence seen with primulin at 0.2 μm (note the expanded *vertical scale* for normalized fluorescence). This inhibition can be reduced by increasing the primulin concentration to 1 μm, indicating that inhibition of primulin binding is competitive. By contrast, the inhibition of fluorescence produced by 0.1 μm MTC cannot be reduced by increasing the concentration of primulin from 0.2 to 1.0 μm, consistent with disaggregation of PHFs by MTC reported previously (10).

A fluorometric assay was developed to measure dissolution of PHFs isolated from AD brain tissue. The addition of PHFs to primulin solution induces a PHF concentration-dependent increase in fluorescence at an excitation wavelength of 420 nm, not seen in the absence of PHFs (Fig. 3*A*), due to the binding of primulin to PHFs (23). A compound based on the primulin scaffold had been used previously to provide the first identification and sequencing of the core Tau unit of the PHF (2), establishing compounds of this type as PHF core ligands. The addition of MTC, previously shown to reverse the proteolytic stability of the PHF core (9), produced concentration-dependent disruption of fluorescence enhancement. Disruption of the primulin-binding can be distinguished from competition at the ligand binding site by its independence from primulin concentration (Fig. 3*B*). This contrasts with competitive ligands, such as thiazine red, where competition can be overcome by increasing the concentration of primulin (Fig. 3*C*). Thiazine red is a ligand that also binds PHFs in AD neurofibrillary tangles (24). Thus, whereas the primulin-binding site remains intact after thiazine red binding, MT destroys the PHF core structure, consistent with previous electron microscopic evidence and reversal of proteolytic stability (9).

We denote the value required for 50% disruption of PHF-primulin binding as the P_50_ value (Table 1). The P_50_ values for MTC, the 1,9-dimethyl-substituted MTC (DMMTC), LMTM, and LMTB were 0.16, 0.18, 0.16, and 0.16 μm, respectively. The structures of these compounds are shown in Fig. 1*B*.

**TABLE 1:** **Inhibition of PHF dissolution, Tau-Tau binding, and Tau aggregation in cells by MTC, DMMTC, LMTM, and LMTB** P_50_, concentration at which there is 50% decrease of PHF-dependent primulin binding; B_50_, concentration at which Tau-Tau binding in a cell-free assay is inhibited by 50%; EC_50_, effective concentration at which aggregation-dependent production of truncated Tau is inhibited by 50% in cells; LD_50_, dose at which 50% of 3T6 mouse cells are killed (as measured by LDH release assay). RxI (therapeutic index) = LD_50_/EC_50_. Data are expressed as the mean (μm) ± S.E. with the number of replicate experiments indicated in parentheses.

Compound	P_50_	B_50_	EC_50_	LD_50_	RxI
	μ*m*	μ*m*	μ*m*	μ*m*	
MTC	0.159 ± 0.013 (4)	195.6 ± 16.1 (10)	0.59 ± 0.04 (73)	65 ± 5 (38)	110
DMMTC	0.180 ± 0.011 (3)	4.3 ± 0.5 (19)	0.041 ± 0.004 (22)	3.5 ± 0.97 (6)	85
LMTM	0.159 ± 0.007 (3)	238.2 ± 74.2 (3)	0.19 ± 0.04 (9)	34 ± 4 (8)	179
LMTB	0.156 ± 0.005 (3)	472.4 ± 22.6 (4)	0.66 ± 0.15 (8)	61 ± 4 (20)	92

##### Inhibition of Tau-Tau Binding through the Repeat Domain in Cell-free Assays

We have previously shown that diaminophenothiazines selectively inhibit Tau-Tau aggregation *in vitro* (9). The inhibitory activities (B_50_) of a range of diaminophenothiazines were compared using this assay (Table 1). DMMTC was almost 50-fold more potent than MTC (*p* < 0.0001). The difference between MTC and LMTM was not statistically significant (*p* = 0.29), but LMTB had lower potency than MTC (*p* < 0.0001).

##### Membrane-targeted Cellular Model of Tau Aggregation

Stable cellular expression of the Tau aggregation domain of the PHF core in eukaryotic cells is difficult due to its inherent toxicity using a number of different vectors and cell lines (25–27) and is insufficient alone to induce aggregation (28). We were able to solve this problem initially by targeting the localization of the truncated Tau fragment to the membrane of the endoplasmic reticulum (ER), using a fusion protein comprising an 18-amino acid signal sequence (SS) (17) at the N terminus of the Tau fragment. 3T6 cells, expressing inducible full-length hTau40, under the control of the *lac* repressor, were co-transfected with vectors containing SS constructs encoding Tau species truncated to variable extents at the N and/or C terminus (SSTau(190–441), SSTau(190–390), and SSTau(296–390); see Fig. 2).

**FIGURE 4.**
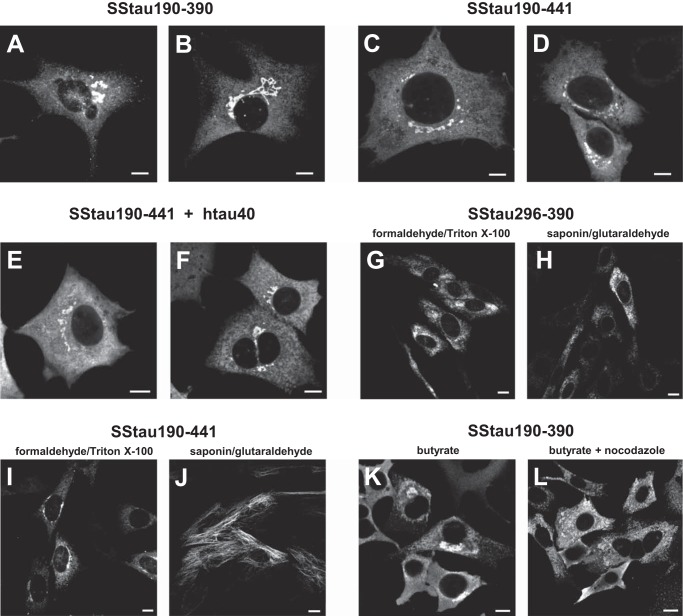
**Immunofluorescence labeling of fibroblasts transfected with ER membrane-targeted constructs.** *A–H*, perinuclear, Tau-immunoreactive deposits were observed in cells transfected with truncated Tau constructs SSTau(190–390) (*A* and *B*), SSTau(190–441) (*C* and *D*), or SSTau(296–390) (*G* and *H*). The deposits were also observed in cells following induction of co-expressed hTau40 (*E* and *F*). Aggregates of SSTau(296–390) were more dispersed into the cytoplasm than the longer construct and remained granular in appearance following saponin treatment (*H*). The Tau from the longer construct, SSTau(190–441), was competent in associating with microtubules (*J*). Perinuclear aggregates formed with SSTau(190–390) (*K*) are distributed throughout the cytoplasm following treatment of nocodazole (*L*), a treatment that both depolymerizes microtubules and disperses the perinuclear stacks of Golgi and ER elements throughout the cell. Cells were treated with butyrate (*A–D* and *G–K*), with butyrate and IPTG (*E* and *F*), or with butyrate plus nocodazole (10 μm for 1 h) (*L*). Cells were fixed with paraformaldehyde followed by extraction with Triton X-100 with the exception of cells that were extracted with saponin and then fixed in glutaraldehyde (*H* and *J*). All cells were labeled using mAb 7/51. *Scale bars*, 10 μm.

Large aggregates, some filamentous in appearance, were observed clustered in the perinuclear area in cells transfected with membrane-targeted constructs. Perinuclear accumulations of Tau immunoreactivity were observed in cells with either SSTau(190–390) (Fig. 4, *A* and *B*) or SSTau(190–441) (Fig. 4, *C* and *D*). Furthermore, the appearance of the Tau was similar for cells expressing SSTau(190–441) in which co-expression of hTau40 had been induced (Fig. 4, *E* and *F*). In contrast, stable transfectants for the membrane-targeted minimum aggregation domain (SSTau(296–390)) expressed lower levels of protein as detected using mAb 7/51. Tau immunoreactivity was observed in a reticular pattern consistent with its association with the ER membrane (Fig. 4*G*), but dense aggregates of Tau were not observed in cells with SSTau(296–390). Tau antibody labeling of the SSTau(296–390)-transfected cells was still observed when cells had been permeabilized with saponin prior to fixation (Fig. 4*H*), indicating that the proteins were oriented on the cytoplasmic surface of membrane organelles. There was no microtubule labeling observed with SSTau(296–390) expression following saponin extraction and using glutaraldehyde as a fixative to preserve microtubules. In contrast, with the SSTau(190–441), microtubules were labeled in the same conditions using mAb 7/51 (Fig. 4*J*). Either the SSTau(190–441) protein is sufficiently long to bind to microtubules while still being attached to the ER in a cytoplasmically oriented direction or the Tau is released into the cytoplasm, where it binds to microtubules. Either way, this contrasts with the behavior of the SSTau(296–390) species.

Paraformaldehyde was found to be a better fixation method for visualization of SSTau aggregates, although this method does not give good fixation of microtubules (29). The perinuclear aggregates seen with SSTau(190–390) (Fig. 4*K*) were dispersed after treatment of cells with nocodazole (Fig. 4*L*). This is again consistent with an association of the Tau with intracellular organelles; nocodazole causes fragmentation and dispersion of the perinuclear Golgi apparatus throughout the cytoplasm (30).

**FIGURE 5.**
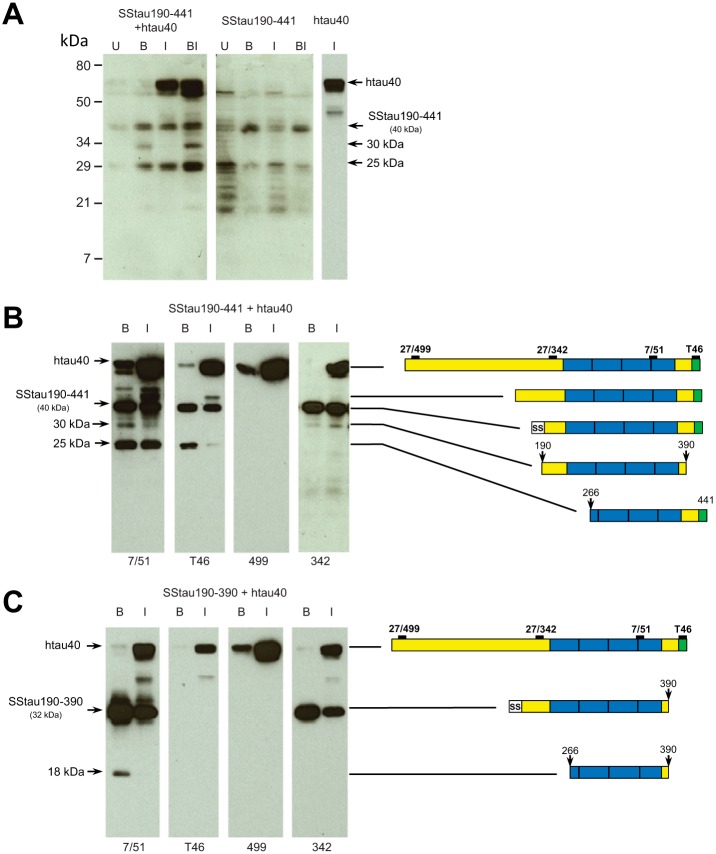
**Aggregation and proteolytic processing of Tau in cells containing SSTau constructs.** *A*, 3T6 cells constitutive for SSTau(190–441) combined with inducible hTau40 (*left*), constitutive for SSTau(190–441) alone (*middle*), or with inducible hTau40 alone (*right*) were analyzed. Cells were treated with 5 mm butyrate, 50 μm IPTG, or both, as indicated, for 24 h before separating by 15% SDS-PAGE and analyzing immunoblots using mAb 7/51 (recognizing an epitope within the microtubule-binding repeat domain). *B* and *C*, epitope analysis of Tau truncation products. Cells with constitutive SSTau(190–441) (*B*) or SSTau(190–390) (*C*) combined with inducible hTau40 were treated with either butyrate or IPTG and analyzed by SDS-PAGE. The identity of the reactive Tau fragments is depicted schematically on the *right*, based upon the apparent mobility and immunoprofile, although the *numbers* for the N and C termini are approximations. *U*, uninduced; *B*, butyrate-induced; *I*, IPTG-induced.

Cell lysates were analyzed by immunoblotting to characterize the Tau products in these stable transfected cell lines (Fig. 5). When SSTau(190–441) (apparent molecular mass ∼40 kDa) was co-expressed in fibroblasts with the inducible hTau40 plasmid, 25-kDa/30-kDa neofragments recognized by mAb 7/51 were generated after induction with butyrate, IPTG, or IPTG plus butyrate. IPTG without butyrate was able to increase the level of the 25-kDa truncation product compared with uninduced cells (Fig. *5**A*, *left*). Increased production of these fragments was not seen when cells expressing SSTau(190–441) alone were induced with butyrate, IPTG, or both (Fig. *5**A*, *middle*). The 25- and 30-kDa neofragments were not seen at all in cells expressing full-length Tau alone (Fig. 5*A*, *right-hand lane*). This indicates that the SSTau(190–441), unlike full-length Tau, can be processed to produce the 25-kDa fragment without induction. Enhanced production of the 25-kDa neofragment following induction with IPTG suggests that there is capture of hTau40 by the SSTau(190–441), which leads to enhanced generation of characteristic truncation products.

The N- and C-terminal extent of these truncation products was partially characterized by analyzing cell lysates with a panel of Tau antibodies recognizing regions of Tau outside the repeat domain. The 18-, 25- and 30-kDa fragments all contain the core aggregation domain of Tau that is recognized by mAb 7/51. The fragments produced from SSTau(190–441) have lost either the N-terminal (25 kDa) or C-terminal domain (30 kDa) (Fig. 5*B*), whereas the 18-kDa fragment produced from SSTau(190–390) lacks both the N- and C-terminal domains (Fig. 5*C*).

Both of the SSTau proteins and the novel truncation products were readily sedimented from cell extracts in the presence of Triton® X-100, indicating the formation of detergent-resistant aggregates. A fraction of the protein sedimented as particles with an apparent *S* value of at least ∼50 S, consistent with aggregation to a complex of ∼1–2 × 10^6^ Da consisting of ∼100-mers of truncated Tau neofragments.

**FIGURE 6.**
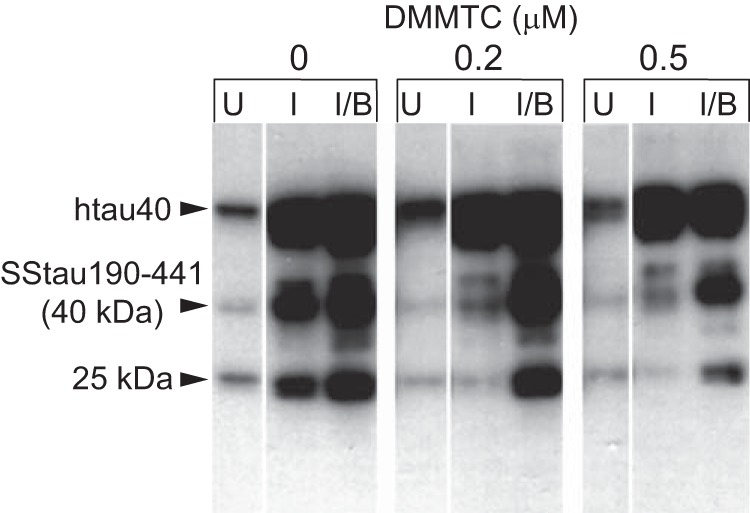
**Effect of DMMTC on Tau aggregation in cells co-expressing constitutive SSTau(190–441) and inducible, full-length hTau40.** Prior to induction with IPTG, and in the absence of DMMTC, there are baseline levels of hTau40 expressed in cells as well as low levels of SSTau(190–441) and its 25-kDa truncation product. When induced with IPTG alone, hTau40 expression was increased together with the amount of the 40- and 25-kDa Tau truncation products. Butyrate induction alone (not shown) or induction with both IPTG and butyrate increased the level of the 25-kDa truncation product. In the presence of DMMTC, the capture of full-length Tau and its conversion to the 25-kDa species was inhibited following either IPTG induction or induction with both IPTG and butyrate. *U*, uninduced; *I*, IPTG-induced; *B*, butyrate-induced.

DMMTC, the most potent MT-related TAI noted above in Table 1, was tested in cells with constitutive expression of SSTau(190–441) and inducible hTau40. In the presence of DMMTC at concentrations of 0.2 and 0.5 μm, the capture of full-length Tau to form the 25-kDa neofragment was inhibited (Fig. 6).

##### Non-membrane-targeted Cellular Model of Tau Aggregation

**FIGURE 7.**
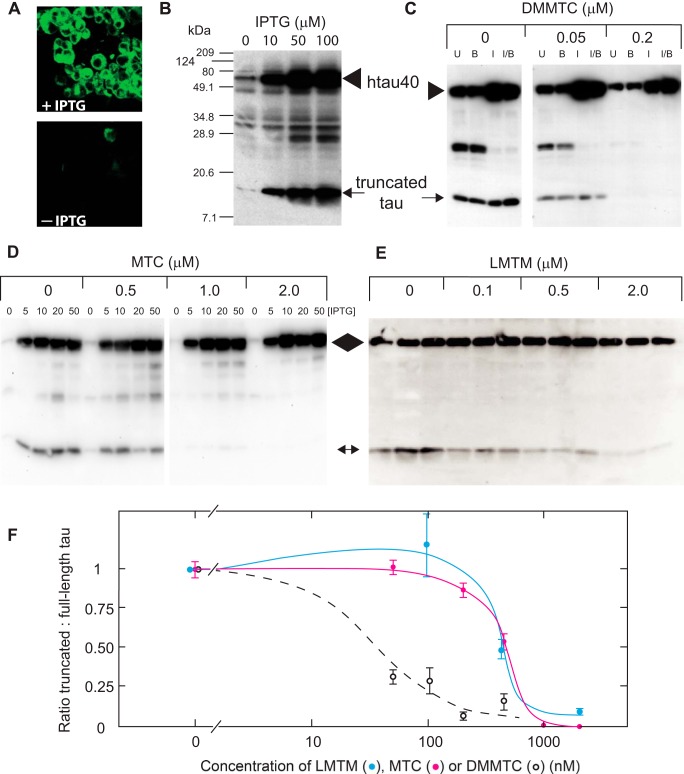
**Cellular model of Tau aggregation and its inhibition by MT.** *A*, fibroblasts, stably transfected with both constitutive truncated Tau(295–391) and uninduced full-length Tau, express both proteins at low levels. Following induction with IPTG, cells showed labeling with primulin, a fluorescent PHF ligand. This was absent for uninduced cells in the *panel below* (− *IPTG*). *B*, SDS-PAGE and immunoblotting of these cells shows that induction of full-length Tau at increasing concentrations of IPTG (*arrowhead*) is associated with increased accumulation of the 12-kDa truncated Tau neofragment (*arrow*). *C*, a dose-dependent inhibition of the generation of the 12-kDa protein from full-length Tau is shown for DMMTC at 0.05 and 0.2 μm, following induction with IPTG, butyrate, or both when compared with uninduced cells. *D*, MTC, tested using a range of IPTG concentrations, also inhibited generation of 12-kDa Tau. *E*, LMTM, tested in triplicate at the same concentrations, but using 10 μm IPTG, was likewise inhibitory over the concentration range 0.1–2.0 μm. The positions of full-length and truncated 12-kDa Tau are indicated by an *arrowhead* and *arrow*, respectively. *F*, inhibition curves are shown for LMTM, MTC, and DMMTC following induction with 10 μm IPTG (mean ± S.E. (*error bars*)). *U*, Uninduced; *I*, IPTG-induced; *B*, butyrate-induced.

The use of membrane targeting produced template-directed, proteolytic neofragments that did not correspond exactly to the PHF core Tau unit. An alternative cellular assay of Tau aggregation, therefore, was developed in which cells expressing inducible full-length hTau40 were stably transfected with pZeo-Tau(295–391), corresponding to the PHF core Tau fragment (Tau(295–391)). The doubly transfected cell line demonstrates the formation of aggregates of Tau that were visualized using the fluorescent PHF ligand primulin only after induction with IPTG (Fig. 7*A*). In these cells, low levels of constitutive, 12-kDa truncated Tau, corresponding to the PHF core Tau unit (Fig. 7*B*, *arrow*) and a low level of full-length Tau (Fig. 7*B*, *arrowhead*), were expressed in the uninduced condition. IPTG-induced expression of full-length Tau led to its conversion to the 12-kDa truncated species (Fig. 7*B*). This did not occur in cell lines in which full-length Tau had been induced in the absence of the co-expressed core Tau fragment (Fig. 5*A*, *third panel*).

**FIGURE 8.**
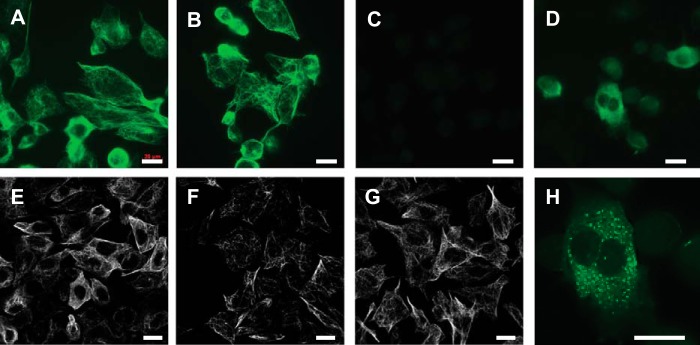
**Immunocytochemical characterization of fibroblasts expressing both Tau(295–391) and inducible full-length Tau.** The cells possessed normal cytoskeletal morphology both before (*A*) and after IPTG induction (*B*), as indicated by the microtubular network labeled for tubulin with mAb YL1/2. Labeling of Tau with mAb 7/51 showed that Tau was only detected after induction (*C*, without IPTG; *D*, with IPTG), and the pattern of staining observed was associated with granular structures. By contrast, cells transfected with inducible full-length Tau, in the absence of truncated Tau(295–391), showed normal microtubular networks after induction, whether labeled with mAbs 27/499 (*E*), 7/51 (*F*), or YL1/2 (tubulin; *G*). A higher magnification of a cell in (*D*) shows the granular staining with mAb 7/51 in the doubly transfected cells following induction of full-length Tau in the absence of microtubule labeling (*H*). Cells were fixed with 0.3% glutaraldehyde with 0.1% Triton X-100. *Scale bars,* 20 μm.

In this cell model, the normal microtubule network was unaffected by the Tau induction process or by constitutive expression of Tau(295–390) (Fig. 8). The tubulin-staining pattern was similar, whether or not cells had been induced with IPTG. By contrast, the Tau labeling with mAb 7/51 was distinct from that observed in cells lacking the truncated Tau species. Expression of induced full-length Tau on its own showed a typical microtubular pattern of staining with mAbs 27/499 and 7/51 (Fig. 8, *E* and *F*). This was not seen for cells co-expressing both full-length and truncated Tau proteins (Fig. 8*D*). The latter showed only a granular staining pattern that is more clearly seen in a cell at greater magnification (Fig. 8*H*). Therefore, full-length Tau induced in the presence of the PHF core unit is processed through an aggregation/proteolysis pathway that prevents association with microtubules.

We have found this model to be reliable and reproducible as a systematic secondary screen for intracellular TAI activity. Results are expressed in terms of reduction in the ratio of the truncated 12-kDa species to induced full-length hTau40, permitting control for different background expression levels. Normalized inhibition is expressed relative to the induced condition in the absence of inhibitor. DMMTC, MTC, LMTB, and LMTM all inhibited conversion of full-length Tau to the 12-kDa species following IPTG induction of full-length Tau (Fig. 7, *C–F*, and Table 1). The EC_50_ values for MTC and LMTB were comparable and not statistically different (*p* = 0.67). By contrast, LMTM was 3-fold more potent than MTC in the cell assay (*p* < 0.0001). The Spearman correlation for EC_50_ *versus* B_50_ values was 0.80, indicating a surprisingly similar ranking of potencies in the cell-free and intracellular assays for TAI activity, despite the considerable differences in absolute values.

Using the *K_d_* value (21.1 ± 2.9 nm) determined *in vitro* for binding of full-length Tau in the aqueous phase to the PHF core fragment in the solid phase (31), and assuming the intracellular concentration of Tau to be 500 nm, *K_i_* values were calculated using the following equation.

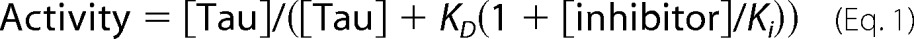
 The *K_i_* values estimated for MTC and DMMTC were 123 nm and 4.4 nm, respectively.

The cellular toxicities (LD_50_ values) for the diaminophenothiazines tested in the cells (Table 1) were used to estimate their therapeutic indices (*i.e.* LD_50_/EC_50_). These indicate that LMTM had the best profile in this series, and DMMTC the poorest profile notwithstanding its higher potency as a TAI in cell-free and cellular assays.

## DISCUSSION

The body of work reported here aims to resolve several fundamental and interrelated problems encountered in the development of the first treatment aiming to block the pathological aggregation of Tau protein as a treatment for AD. We recently reported that the treatment effects of MTC tested in a phase 2 clinical trial of mild or moderate AD were highly determined by a combination of redox processing in the gut and the ability to absorb MT in the presence of food (11). Although MTC dosed at a level of 138 mg MT/day was found to arrest progression of AD on a range of clinical and imaging outcomes, a dose of 228 mg MT/day had lower than expected efficacy on the same outcomes. This was due to dose-dependent limitations in the ability to absorb MT when dosed in the oxidized cationic form as MTC. Pharmacokinetic studies in transgenic Tau mice and humans have shown that dosing of MT in the stable reduced form solves the absorption problem (11). One important question, therefore, is to determine whether reduced forms of MT retain TAI activity in a range of assay systems.

A further closely related issue is to determine whether the clinical benefit seen with MT at 138 mg/day is plausibly due to TAI activity or whether it is due to other potential activities that have been proposed (reviewed in Ref. 32). This in turn depends on defining the MT concentration required for dissolution of native PHFs and the *K_i_* for intracellular TAI activity. These values can then be compared with estimates of the minimum brain concentration of MT required for clinical efficacy in humans (0.18 μm) (11) and the minimum brain concentration required for reversal of behavioral deficits and reduction of Tau aggregation pathology in transgenic mouse models (0.13 μm) (12).

We first report the synthesis and physico-chemical characterization of stabilized diprotic acid salts of LMT that we have used in pharmacokinetic (11) and transgenic animal studies (12). We refer to them as the novel chemical entity class, LMTX®. X-ray crystal structure determinations of LMTM demonstrate that the nitrogen atoms at positions 3 and 7 have tetrahedral geometry. The protonation of these atoms accounts for both the geometry and the stability of LMTM and distinguishes the latter from MW, in which the corresponding nitrogen atoms are in a trigonal pyramidal geometry and not protonated. Synthesis of MW has to be performed under an inert atmosphere because it rapidly oxidizes on exposure to air. This property underlies the use of MW as an oxygen sensor to monitor the security of sealed components, where it is converted to the blue MT cation once such seals are broken (33). In contrast, LMTM can be manufactured in bulk without the need for deoxygenation and remains stable for at least 2 years when open to an air atmosphere. Thus, LMTM represents a new chemical entity that is distinct from both MTC and MW. LMTM is highly soluble and exists as a single polymorph, in contrast to MTC, which is far less soluble and demonstrates heterogeneous polymorphism (13).

We have reported previously, on the basis of electron microscopic and biochemical evidence, that MTC is able to dissolve proteolytically stable PHFs and convert the constituent Tau protein into a form that is readily susceptible to proteases (9). We now report a fluorescence assay in which it is possible to quantify this activity and to distinguish PHF disruption from competitive ligand-binding activity. The P_50_ values for this activity for the range of MT derivatives we have studied are ∼0.16 μm. The use of primulin as a sensor to report the aggregated state of the core PHF is based on our previous evidence that primulin is the principle tangle-binding constituent of crude commercial thioflavin-S preparations (23) and the use of a compound based on the primulin scaffold to provide the first identification and sequencing of the core Tau unit of the PHF (2). The enhanced fluorescence is concentration-dependent with respect to PHFs and is not seen in the absence of PHFs. We further distinguish reduction of primulin binding to PHFs that is due to competing ligand activity from disruption of PHF integrity by TAIs. The P_50_ values reported here therefore define the concentration of TAI required for dissolution of native PHFs isolated from AD brain.

In the *in vitro* Tau-Tau binding assay, in which truncated Tau is in a partially aggregated state, the B_50_ values for MTC and LMTM did not differ significantly; LMTB had a 2.4-fold lower potency. DMMTC was found to have a 55-fold higher potency than MTC in the *in vitro* Tau-Tau binding assay. It did not, however, differ in potency in terms of PHF disruption. Furthermore, the B_50_ values ranged between 24- and 3,000-fold higher than the corresponding P_50_ values. These discrepancies illustrate the importance of defining an assay for TAI activity that has disease relevance. Differences in TAI activity between PHFs and the *in vitro* assay are most likely due to differences in the state of aggregation of the core Tau domain *in vitro* compared with native PHFs formed within the brain and to difficulties in controlling its autoaggregation *in vitro*. An important corollary is that it is not possible to define the concentrations required for TAI activity *in vivo* based solely on Tau aggregation or polymerization assays *in vitro* (34–38).

The difficulties encountered in controlling Tau aggregation *in vitro* and the discrepancies with respect to actual PHFs from AD brain suggest the need to model the process in a more physiological setting within a living cell. This has proved difficult to achieve directly by simple overexpression, even when using inducible expression vectors, due to the inherent cytotoxicity of the core PHF Tau fragment. Although the exact form of Tau that is toxic in AD and the cellular location of such toxicity remain uncertain (25), we have observed toxicity of both truncated Tau and PHF-Tau in Lipofectin transfection models (39). We now report the development of two novel cell models in which it has been possible to induce Tau aggregation through the repeat domain and to generate aggregation-dependent truncation products within the physiological milieu of the cell. We have used fibroblast cell lines that lack endogenous Tau protein (40). In the first model, we have targeted the expression of truncated Tau to the ER membrane, where, at low levels, it is capable of initiating the capture and aggregation of full-length human Tau. Using truncated Tau proteins that incorporate the repeat domain as initiators, we have demonstrated that cells are able to generate two families of neofragments restricted to either three or four repeats. The oligomers that are formed are the equivalent of 100-mers of truncated Tau neofragments. The combined expression of the truncated initiator species and full-length Tau protein therefore leads to the processing of the latter to form increased levels of characteristic truncated neofragments of Tau.

The generation of neofragments by directing truncated Tau expression to the ER membrane is interesting in the light of evidence that early amorphous aggregates of Tau in AD are associated with mitochondria and ER membranes (41). Furthermore, there is increased contact between mitochondria and rough ER membranes in JNLP3 mice that express P301L mutant Tau, and accumulation of Tau was identified in ER membrane-enriched fractions from both these transgenic mice and AD brains (42).

Whereas the SS-models demonstrate the principle of prion-like processing of Tau protein, the neofragments generated are larger than the PHF core domain that they all contain. We found that, provided constitutive expression of the PHF core fragment is maintained at very low levels, long term cell viability is possible. These low levels do not, however, permit aggregation to be studied. When such cells were co-transfected with inducible, full-length four-repeat Tau, it was possible to induce Tau aggregation and template-directed truncation of full-length Tau in a controlled manner. The neofragment generated in a concentration-dependent manner with respect to induced full-length Tau is simply the core Tau unit itself. This now confirms within the physiological milieu of the cell the model of propagation of Tau aggregation through the repeat domain that we first reported on the basis of data in a cell-free system (9). The repeat domain has the ability to define a template-directed truncation of full-length Tau to reproduce and amplify the proteolytically stable species characteristic of the PHF core in AD (2, 9).

In this model, Tau aggregation can be demonstrated only after induction of full-length Tau. Furthermore, whereas induction of full-length Tau in the absence of the PHF core fragment permits binding to microtubules, this does not occur in cells co-expressing the PHF core fragment. Instead, newly expressed full-length Tau is preferentially directed to the aggregation/truncation pathway. This is consistent with *in vitro* data that we have reported previously showing that the binding affinity for Tau-Tau binding is substantially higher than for Tau-tubulin binding, although both occur through the repeat domain (31). This may help explain the almost complete redistribution of the Tau protein pool from microtubule-bound to aggregated forms that is characteristic of AD (14) and indicates that phosphorylation is not required to account for loss of microtubule binding in AD (43, 44).

We have further demonstrated that template-directed truncation can be inhibited in both cellular models by substances shown to be TAIs *in vitro*. Indeed, the Spearman correlation for EC_50_ *versus* B_50_ values was 0.80, indicating an approximately similar ranking of potencies in the cell-free and intracellular assays for TAI activity. This is despite the fact that the cell assay requires an additional cell entry step. These data support the aggregation dependence of template-directed truncation. It is again important to note that the only exogenous manipulation required to induce aggregation and prion-like processing is to turn on the expression of full-length Tau in the presence of a truncated Tau binding partner. The aggregation of Tau through the repeat domain does not require phosphorylation (45) or the formation of covalent bonds, as has been proposed (46). The fact that *K_i_* values can be calculated using a standard kinetic model implies that Tau-Tau binding and its inhibition are concentration-dependent, competitive, saturable, and reversible within the cell milieu, inconsistent with the formation of covalent bonds (46).

**FIGURE 9.**
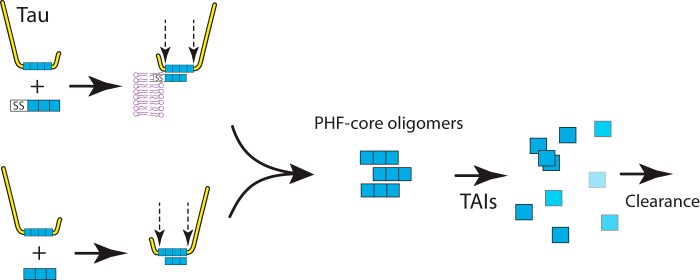
**Schematic representation of Tau aggregation in cell models.** Tau aggregation requires the presence of both a truncated Tau fragment (either with or without the SS) and a full-length molecule of Tau protein. The combination of these, followed by proteolysis of larger Tau, restricted to the N and C termini, leads to the accumulation of truncated PHF core Tau oligomers. Formation of the oligomers needed for template-directed truncation can be blocked and their clearance can be enhanced by diaminophenothiazine TAIs.

Our findings with the two cell models are consistent with a pathway of events summarized schematically in Fig. 9. (i) Aggregation of Tau is seeded initially either by the membrane-localized truncated Tau or by truncated Tau itself interacting with full-length Tau. (ii) Cleavage of the N-terminal signal peptide and/or the N and C termini of full-length Tau (iii) leaves Tau oligomers in the cytoplasm. (iv) These oligomers allow further capture of full-length Tau that enters the proteolytic processing pathway. (v) Oligomers are susceptible to disaggregation by TAIs, allowing for their subsequent clearance from the cell.

The cellular evidence for Tau aggregation being seeded by truncated Tau (47, 48) has been confirmed independently in transgenic rat studies (49, 50). In the latter studies, insoluble Tau aggregates formed in the brain consist of both transgenic human truncated Tau and endogenous rat Tau in a 1:1 ratio. This confirms, as predicted by the earlier *in vitro* data (9) and the cell-based data described here, that truncated Tau coopts normal Tau into a pathological aggregation pathway leading ultimately to aggregates typically formed within neurons in AD. This suggests that truncated Tau oligomers are sufficient to drive neurofibrillary degeneration in the presence of full-length Tau. Truncated Tau oligomers are also important for the transmission of Tau pathology between neurons that has been established in recent years (47, 48, 51).

Based upon the cell models described here, we have gone on to create transgenic mice to demonstrate that truncated Tau targeted to the ER membrane also leads to Tau aggregation *in vivo* (52). The mice show evidence of neuroanatomical spread of Tau aggregation pathology and amplification with age that resembles the Braak staging of AD. Furthermore, MTC and LMTX®, which we have shown to be active inhibitors of Tau aggregation, reduce Tau pathology and reverse the cognitive behavioral deficits observed in these mice (12). Treatment with these compounds also reduced Tau pathology and reversed behavioral deficits in a further transgenic Tau model for the frontotemporal lobar degeneration spectrum based on full-length mutant Tau (12).

Since we described the selective inhibition of Tau-Tau binding by MTC (9), several other Tau polymerization inhibitors have been reported. These include bis-thiacarbocyanine (36, 53), Congo red derivatives and anthoquinones (37), 2,3-di(furan-2-yl)-quinoxalines (35), phenylthiazolyl-hydrazide (54), polyphenols and porphyrins (38), oleocanthal (55), rhodanines (56), and cyanin dyes (34). Some of these are toxic, and their potency either as disruptors of PHFs *in vitro* or their activities in more physiological intracellular conditions remains largely uncharacterized. The activity of some of these compounds has been tested in neuroblastoma (57) or *Caenorhabditis elegans* (58) models in which mutant Tau proteins have been used. Because these mutations do not occur in AD, the relevance of such models to the aggregation found in AD is unknown. Testing such compounds in transgenic animal models is labor- and time-intensive, making the cell models we have described a useful intermediate platform for lead selection and for optimization for TAI activity. We illustrate this utility with DMMTC. Although DMMTC is a highly potent TAI in both cell-free and cellular assays for Tau aggregation, it is also a substantially more toxic compound than MTC. LMTM, although having lower potency than MTC in cell-free and cell-based assays, nevertheless has a better therapeutic index and has better bioavailability *in vivo* (11).

Finally, the cell-based models permitting TAI activity to be measured in a physiological milieu provide a means of estimating the intracellular *K_i_* and linking this to the concentration required for TAI activity in the human brain in AD. Thus, the intracellular *K_i_* for activity of MT at the Tau aggregation site is 0.12 μm. This value is close to the P_50_ value for disaggregation of PHFs isolated from AD brain (0.16 μm). These concentrations are both close to the estimated steady state trough brain concentration of MT and its pharmacologically active metabolites (0.18 μm) at the minimum effective dose for treatment of AD and the minimum brain concentration (0.13 μm) required for beneficial effects on behavior and pathology in Tau transgenic mice. The cell models described here therefore offer potential to investigate the process of aggregation in a living system, the mechanism of action of TAIs, and to optimize them with a view to further clinical development. Because the P_50_ and *K_i_* values are both close to the brain concentration required for therapeutic efficacy, the data do not as yet enable us to distinguish whether it is the ability of MT compounds to disaggregate PHFs and related oligomers, their ability to prevent their aggregation, or a combination of these that is critical for clinical efficacy of TAI therapy in AD and related disorders.

## Supplementary Material

Supplemental Data

## Footnotes

3 The abbreviations used are:

AD: Alzheimer disease
DMMTC: dimethyl methylthioninium chloride
ER: endoplasmic reticulum
IPTG: isopropyl thio-β-d-galactoside
LMT: leucomethylthioninium
LMTB: LMT-dihydrobromide
LMTM: LMT-dihydromesylate
MT: methylthioninium
MTC: methylthioninium chloride
MW: methylene white
PHF: paired helical filament
SS: signal sequence
TAI: Tau aggregation inhibitor.
