# Supplementary material for: Cellular Models of Aggregation-dependent Template-directed Proteolysis to Characterize Tau Aggregation Inhibitors for Treatment of Alzheimer Disease
Source: J Biol Chem. 2015 Mar 10;290(17):10862–75. doi: 10.1074/jbc.M114.616029 (PMC4409250; doi:10.1074/jbc.M114.616029)
Supplement: Supplemental Data [file supp_290_17_10862__index.html]

Cellular Models of Aggregation-Dependent Template-Directed Proteolysis to Characterize Tau Aggregation Inhibitors for Treatment of Alzheimer's Disease — Cellular Models of Aggregation-dependent Template-directed Proteolysis to Characterize Tau Aggregation Inhibitors for Treatment of Alzheimer Disease — Cell Models of Tau Aggregation — Supplemental Data 

# Cellular Models of Aggregation-dependent Template-directed Proteolysis to Characterize Tau Aggregation Inhibitors for Treatment of Alzheimer Disease

## Supplemental Data

**Files in this Data Supplement:**

- Supplemental data (.pdf, 173 KB) - Supplementary data including: experimental, 2 tables and 2 figures.
