# Supplementary material for: Cellular Models of Aggregation-dependent Template-directed Proteolysis to Characterize Tau Aggregation Inhibitors for Treatment of Alzheimer Disease
Source: J Biol Chem. 2015 Mar 10;290(17):10862–75. doi: 10.1074/jbc.M114.616029 (PMC4409250; doi:10.1074/jbc.M114.616029)

Cellular Models of Aggregation-Dependent Template-Directed Proteolysis to Characterize Tau Aggregation Inhibitors for Treatment of Alzheimer's Disease *[S]

Charles R. Harrington[1,†,‡,$], John M. D. Storey[‡,§,$], Scott Clunas[§,$], Kathleen A. Harrington[†], David Horsley[†,$], Ahtsham Ishaq[§,$], Steven J. Kemp[§,$], Christopher P. Larch[§,$], Colin Marshall[§,$], Sarah L. Nicoll[§,$], Janet E. Rickard[†,$], Michael Simpson[§,$], James P. Sinclair[§,$], Lynda J. Storey[§,$], and Claude M. Wischik[1,†,‡,$]

From the [†] School of Medicine and Dentistry, University of Aberdeen, Aberdeen AB25 2ZP, UK; [‡] TauRx Therapeutics Ltd., Singapore 068805, and the [§] Department of Chemistry, University of Aberdeen, Aberdeen AB24 3UE, UK

[°] Running title: *Cell models of tau aggregation*

[1] To whom correspondence should be addressed: Charles R. Harrington, School of Medicine and Dentistry, University of Aberdeen, Foresterhill, Aberdeen AB25 2ZP, UK, Tel: (+44) 1224 438563; Fax: +44 1224 555173; E-mail: c.harrington@abdn.ac.uk

Claude M. Wischik, School of Medicine and Dentistry, University of Aberdeen, Foresterhill, Aberdeen AB25 2ZP, UK, Tel: (+44) 1224 438550; Fax: +44 1224 555173; E-mail: cmw@taurx.com

Supplemental data

(Experimental; 2 Tables; 2 Figures)

Experimental

Synthesis of *N,N,N′,N′*-tetramethyl-10*H*-phenothiazine-3,7-diamine derivatives.

10-Acetyl-*N,N,N′,N′*-tetramethyl-10*H*-phenothiazine-3,7-diamine was synthesized and used as the starting material for the synthesis of *N,N,N′,N′*-tetramethyl-10*H*-phenothiazine-3,7-diaminium dibromide (leucomethylthioninium dihydrobromide; LMTB) and *N,N,N′,N′*-tetramethyl-10*H*-phenothiazine-3,7-diaminium bismethanesulphonate (leucomethylthioninium dihydromesylate; LMTM). The structures of LMTM and LMTB are included in Fig. 1*B*.

Synthesis of 10-acetyl-*N*,*N*,*N′*,*N′*-tetramethyl-10*H*-phenothiazine-3,7-diamine.

MTC (150.0 g, 0.387 mol inclusive of 17.5% w/w H_2O) was added to a three-neck round bottomed flask under a N_2 atmosphere. Acetonitrile (300 ml) was added, followed by triethylamine (83.9 ml, 0.601 mol) and hydrazine monohydrate (11.7 ml, 0.241 mol). The mixture was heated to 70 ^{o}C and held at this temperature for 1 hour, after which the temperature was reduced to 50 ^{o}C and acetic anhydride (227.6 ml, 2.406 mol) was added over 30 min. The mixture was then heated to 95 ^{o}C and held for 2 h, after which the temperature was reduced to 60 ^{o}C and water (345 ml) was added over 30 min. The resulting slurry was cooled to and held at 3 ^{o}C for 2 h. The solid was collected by filtration and washed with water (3 x 300 ml) followed by drying under vacuum (10 mmHg) for 18 h at 50 ^{o}C to give the title compound as a grey solid; mp 179-181 °C (93.01 g, 71%).

^{1}H NMR (DMSO-d_6, 400 MHz, ppm): δ 2.05 (3H, s, $COCH_3$), 2.89 (12H, s, $2N(CH_3)_2$), 6.66 (2H, dd, *J* = 8.6 & 2.4 Hz, 2ArH), 6.74 (2H, d, *J* = 2.4 Hz, 2ArH), 7.33 (2H, d, *J* = 8.6 Hz, 2ArH); ^{13}C NMR ($CDCl_3$, 100 MHz, ppm): δ 23.0 ($\mathit{C}H_3$), 40.6 ($2N\mathit{C}H_3$), 110.7 (2Ar*C*), 110.9 (2Ar*C*), 127.0 (2Ar*C*), 128.9 (2ArC), 148.9 (2ArC), 170.1 (C=O); FTIR, diamond, cm^{-1}: ν_{max} 1657 (m), 1592 (m), 1496 (m), 1322 (m), 1008 (m), 857 (m), 806 (s); MS (TOF ESI positive mode): m/z calc'd for $[M]^+$, $(C_{18}H_{21}N_3OS)^+$: 327; Found: 284 (100%, $[M – OAc]^+$), 328 (67%, $[M + H]^+$), 350 (37%, $[M + Na]^+$).

Synthesis of *N*,*N*,*N′*,*N′*-tetramethyl-10*H*-phenothiazine-3,7-diaminium dibromide (LMTB).

10-Acetyl-*N,N,N′,N′*-tetramethyl-10*H*-phenothiazine-3,7-diamine (300 g, 0.917 mol) was slurried in water (375 ml) and hydrobromic acid (690 ml, 48%, 6.13mol) was added over 5 min. The mixture was heated at 80 °C for 2 h then cooled and held at 5 °C for 1 h. The solid that

precipitated from solution was collected by filtration. This was then dissolved in water (923 ml) and hydrobromic acid (92.3 ml, 48%, 0.82 mol), before being heated at 90 °C for 20 min. The mixture was cooled to 40 °C and a second aliquot of hydrobromic acid (92.3 ml, 48%, 0.82 mol) was added. The mixture was then cooled and held at 5 °C for 1 h to crystallise the product from solution. The product was collected by filtration and dried at 40 °C under reduced pressure (10 mmHg) to give the title compound as pale yellow-green crystals; mp 270-280 ^{o}C with decomposition (335 g, 82%) (99.7% pure by HPLC analysis).

^{1}H NMR (D_2O, 400 MHz, ppm): δ 3.07 (12H, s, $N(CH_3)_2$), 6.63 (2H, d, *J* = 8.8 Hz, Ar–H), 7.08 (2H, d, *J* =8.8 Hz, Ar-H), 7.09 (2H, s, Ar-H); ^{13}C NMR (D_2O, 100 MHz, ppm): δ 46.3 ($2N(CH_3)_2$), 115.2 (2Ar-C), 118.1 (2Ar-C) 118.4 (2Ar-C), 120.0 (2Ar-C), 136.8 (2Ar-C), 142.3 (2Ar-C); FTIR, KBr disc, cm^{-1}: ν_{max} 3459 (b), 3237 (m), 3194 (m), 3119 (m), 3048 (m), 3013 (m), 2954 (m), 2877 (m), 1487 (s), 1405 (s), 1316 (s), 1188 (m), 1125 (m); Elemental Analysis (% w/w): Theoretical C = 42.97, H = 4.73, N = 9.40, Br = 35.73; Found C = 42.65, H = 4.66, N = 9.30, Br = 35.69; MS (ESI positive mode): m/z calc'd for $[M]^+$ ($C_{16}H_{19}N_3S$): 285; Found: 285 (100%, $[M]^+$), 286 (45%, $[M+H]^+$).

Synthesis of *N,N,N′,N′*-tetramethyl-10*H*-phenothiazine-3,7-diaminium bismethanesulphonate (LMTM).

10-Acetyl-*N,N,N′,N′*-tetramethyl-10*H*-phenothiazine-3,7-diamine (100.0 g, 0.305 mol) was added to a three-necked round bottomed flask equipped with a condenser, dropping funnel and a thermometer. Methanesulphonic acid (68.5 ml, 70%, 0.672 mol) and H_2O (10 ml) was added, and the mixture heated to and held at 78 °C for 2.5 h. The reaction progress was monitored by TLC (1:1 EtOAc/40:60 petrol; R_f: 0.75). Upon completion, the mixture was cooled to 50 °C

whereupon ethanol (500 ml) was added drop-wise over 2 h. The mixture was then cooled to 5 °C for 1 h and the light green solid that precipitated was collected by filtration, washed with acetonitrile (3 x 100 ml) and dried at 38 ^{o}C for 18 h; mp 271 ^{o}C (130.0 g, 89%).

^{1}H NMR (CD_3OD, 600 MHz, ppm): δ 2.71 (6H, s, $2CH_3SO_3$), 3.21 (12H, s, $2N(CH_3)_2$), 6.75 (2H, d, *J* = 8.8 Hz, 2ArH), 7.22 (2H, d, *J* = 2.8 Hz, 2ArH), 7.24 (2H, dd, *J* = 8.8 & 2.9 Hz, 2ArH); ^{13}C NMR (CD_3OD, 150 MHz, ppm, MeOH-d_4): δ 38.2 ($2CH_3SO_3$), 45.9 ($2NMe_2$), 115 (2ArCH), 118.2 (2ArCH), 118.7 (2ArC), 119.9 (2ArCH), 137.1 (2ArC), 142.8 (2ArC); FTIR, KBr disc, cm^{-1}: ν_{max} 3430 (b), 3014 (m), 2649 (m), 1614 (m), 1487 (s), 1318 (s), 1199 (s), 1059 (s), 823 (s); Elemental Analysis (% w/w): Theoretical C = 45.26; H = 5.70; N = 8.80; S = 20.14; Found C = 45.19; H = 5.53; N = 8.76; S = 19.84; Exact mass (TOF ES^{+} m/z): calc'd for $[M]^{+}$ ($C_{16}H_{19}N_3S$): 285.129970, Found = 285.131292; UV (λ_{max} nm): 255.

Synthesis of Methylene White (MW). MTC (17.58% w/w H_2O) (9.48 g, 24.5 mmol) and acetonitrile (50 ml) were loaded under a positive flow of argon into a glass ampoule fitted with a Young's tap. Hydrazine monohydrate (2.70 g, 54.0 mmol) was added cautiously, also under a positive flow of argon, and the reaction mixture was heated to 50 °C. After 3 h at 50 °C the reaction mixture was cooled to 5 °C. The resulting brown solution was filtered away from the solid by filter cannula using a positive flow of argon. The green solid left behind was washed with cold (5 °C) acetonitrile (1 x 30 ml) using a filter cannula. The green solid was then dried *in vacuo* for 10 mins and tetrahydrofuran (THF) (20 ml) was added. The orange THF solution was filtered away from insoluble residues by filter cannula into a second argon filled ampoule and subsequent removal of the THF *in vacuo* gave the title compound as a pale yellow solid that was stored under argon.

^{1}H NMR (THF-d_8, 400 MHz, ppm): δ 2.72 (12H, s, $2N(CH_3)_2$), 6.34-6.41 (6H, m, Ar-H), 6.79 (1H, s, NH); ^{13}C NMR (THF-d_8, 100 MHz, ppm): δ 41.6 (4C, $N(CH_3)_2$) 112.4 (2Ar-C); 113.3 (2Ar-C); 115.2 (2Ar-C); 119.2 (2Ar-C); 135.4 (2Ar-C); 147.5 (2Ar-C). FT-IR (Nicolet IR100 Diamond, cm^{-1}): 3392 (w), 2791 (br), 1601 (w), 1500 (s) 1477 (s), 1303 (s), 1220 (s), 796 (s); MS (ESI positive mode): m/z calc'd for $[M]^+$ ($C_{16}H_{19}N_3S$): 285; Found = 143 $[M+H/2]^{2+}$ 100%, 285 $[M]^+$ 72%, 286 $[M+H]^+$ 70%. UV (λ_{max} nm): 260.

Table S1. Metal content analyses for three batches of LMTM, synthesised to semi-commercial scale, and compared with the European Pharmacopoeia (EP) 5.4 limits for heavy metals in methylthioninium chloride (Council of Europe (2006) Methylthioninium chloride. European Pharmacopoeia Edn. 5.4:pp. 3977-3979).

**Metal**	**Maximum content (ppm) in EP 5.4**	**Metal content in LMTM batches (ppm)**		
		**Batch 1 (86 kg)**	**Batch 2 (90 kg)**	**Batch 3 (79 kg)**
Copper	300 ppm	< 10	< 10	< 10
Zinc	100 ppm	< 10	< 10	< 10
Aluminium	100 ppm	< 10	< 10	< 10
Cadmium	1 ppm	< 0.1	< 0.1	< 0.1
Chromium	100 ppm	< 10	< 10	< 10
Tin	10 ppm	< 1	< 1	< 1
Iron	200 ppm	< 10	< 10	< 10
Manganese	10 ppm	< 1	< 1	< 1
Mercury	1 ppm	< 0.1	< 0.1	< 0.1
Molybdenum	10 ppm	< 1	< 1	< 1
Nickel	10 ppm	1	1	< 1
Lead	10 ppm	< 1	< 1	< 1

Table S2. Stability data for a representative LMTM sample stored at 30 °C and 65% relative humidity. Assay for potency and water content are expressed as % mean values by wt. XRPD was tested initially and after 12 and 24 months. ND, not detected; RH, relative humidity; MT, methylthioninium ion; acetyl MT, 10-Acetyl-*N,N,N′,N′*-tetramethyl-10*H*-phenothiazine-3,7-diamine; --, not tested (Azure B was never detected at the 1-month time-point in batches and was only tested thereafter at the 24-months).

Test	Initial sample	Storage condition (30°C / 65% RH) (months)						
		1	3	6	9	12	18	24
Assay (mean)	99.7%	99.1%	99.8%	99.8%	100.0%	99.8%	99.2%	99.4%
Related substances								
Leuco-Azure B	ND	ND	ND	ND	ND	ND	ND	<0.05%
MT	0.63%	0.75%	0.50%	0.62%	0.74%	0.58%	0.95%	0.55%
Acetyl MT	ND	ND	ND	ND	ND	ND	ND	ND
Azure B	--	--	--	--	--	--	--	ND
Total impurities	0.63%	0.75%	0.50%	0.62%	0.74%	0.58%	0.95%	0.55%
Water content (mean)	0.04%	0.07%	0.14%	0.11%	0.04%	0.04%	0.13%	0.07%
XRPD	Form A	--	--	--	--	Form A	--	Form A

Fig. S1. The Raman spectrum of LMTM form A showing a significant background from fluorescence. However, a number of sharp signals are observed and can be used for the identification of the crystalline form. The instrument used was Bruker RF100. Nd:YAG 1064 nm excitation, 50 mW laser power, Ge-detector, 128 scans, range 50-3500 cm-1, 2 cm-1 resolution; with an aluminium sample holder.

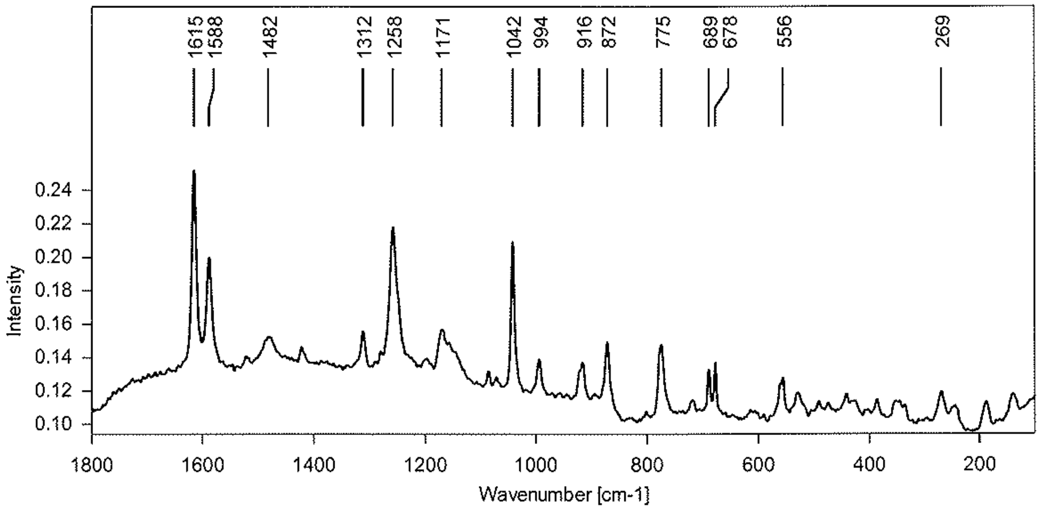

Fig. S2. X-ray diffraction pattern (XRDP) analysis of LMTM polymorphic form A. (Bruker D8 Advance, Cu Ka radiation (l =1.54180 Å), 40 kV/40 mA Lynex Eye detector, 0.02° step size in 2q, 37s per step, 2.5°-50° 2q scanning range). The samples were prepared on silicon single crystal sample holders with 0.1- or 1.0-mm depth without any special treatment other than the application of slight pressure to get a flat surface. All samples were rotated during the measurement.

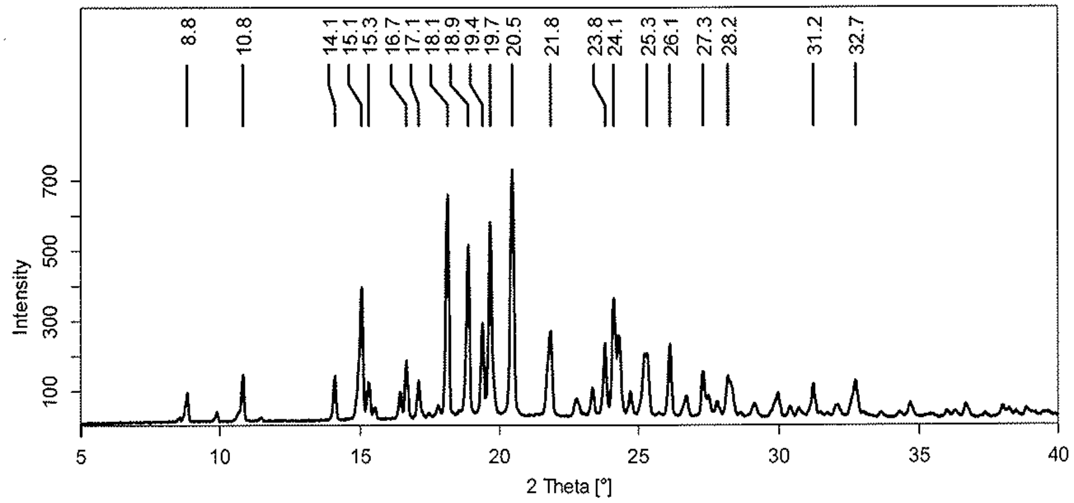

Supplement: Supplemental Data [file supp_M114.616029_jbc.M114.616029-1.pdf]
